# A Case Report of a Multisystemic Immune‐Related Adverse Event Caused by Sintilimab in Combination With Thymosin Alpha‐1

**DOI:** 10.1002/ccr3.72025

**Published:** 2026-02-08

**Authors:** Ting Li, Bao‐Liang Wu, Cai‐Long Yu, Liang‐Yan Jin

**Affiliations:** ^1^ Department of Clinical Pharmacology Hangzhou Xixi Hospital Hangzhou China; ^2^ Department of Medical Imaging Hangzhou Xixi Hospital Hangzhou China

**Keywords:** combination immunotherapy, immune‐related adverse events, sintilimab, thymosin alpha‐1

## Abstract

To report a case of a nasopharyngeal carcinoma patient who developed multisystem immune‐related adverse events (irAEs) after treatment with sintilimab (PD‐1 inhibitor) in combination with thymosin alpha‐1 (Tα1). The patient presents with high fever, rash, interstitial pulmonary edema, and multiple organ failure. By analyzing the course of treatment and regression, the risks and management strategies of combining immune checkpoint inhibitors (ICIs) with immunomodulators are discussed in the light of the literature. This highlights the need for clinical vigilance against possible immune overactivation triggered by combination therapy.

## Key Clinical Message

Combination therapy with immune checkpoint inhibitors and immunostimulants like thymosin alpha‐1 may synergistically overactivate the immune system, leading to severe multisystem immune‐related adverse events. Clinicians should exercise extreme caution with such combinations, especially in patients with a history of prior immune‐related toxicity.

**Abbreviations**
ALT: alanine aminotransferase
AST: aspartate aminotransferase
CRP: C‐reactive protein
CTLA‐4: cytotoxic T lymphocyte‐associated antigen‐4
EGFR: the anti–epidermal growth factor receptor
GGT: gamma‐glutamyl transferase
ICIs: immune checkpoint inhibitors
irAEs: immune‐related adverse events
PCT: procalcitonin
PD‐1: programmed death‐1
PLT: platelet count
PT: prothrombin time
PT activity: prothrombin time activity
SAA: serum amyloid A
Tα1: thymosin α1
WBC: white blood cell count

## 1 Introduction

Immune checkpoint inhibitors (ICIs), such as the programmed death‐1 (PD‐1) inhibitor sintilimab, have become cornerstone therapies in oncology [1]. However, while ICIs modulate the immune response to target and kill tumor cells, over‐activation of immune cells can also lead to autoimmune damage, which can result in immune‐related adverse events (irAEs) involving multiple systems, such as the skin, liver, lungs, and endocrine system [2] Severe, life‐threatening irAEs have also been reported with sintilimab‐based regimens [3].

Thymosin alpha‐1 (Tα1) is an immunomodulatory peptide known to play critical roles in T cell maturity and differentiation [4]. Enhancement of immunity by promoting T cell differentiation and maturation, natural killer cell, dendritic cell activation, and proinflammatory cytokine release. It is now widely used in diseases including viral, bacterial and fungal infections, immune disorders, immunodeficiencies, and tumors. Several studies have explored thymic peptides as immune modulators, alone or in combination, for the treatment of certain types of cancer, such as melanoma, hepatocellular carcinoma, and lung cancer [5, 6].

While ICIs have transformed the way cancer patients are treated, there are still relatively low response rates and certain safety concerns. Given the role of Tα1 in regulating cellular immunity and the exceptional safety profile demonstrated over decades of clinical use, more and more studies are exploring the use of the two in combination to improve antitumor efficacy and reduce immune‐related toxicity [7]. However, our patient developed life‐threatening multisystemic immunological adverse events such as high fever, rash, interstitial pulmonary edema, and coagulation disorders after treatment with sintilimab in combination with Tα1. To analyze the underlying mechanisms and to explore the protocols and monitoring strategies for the combined use of ICIs and Tα1.

## 2 Case History/Examination

A 29‐year‐old male (weight 84 kg) was diagnosed with stage cT4N3M0 nasopharyngeal carcinoma in April 2024.

On Day 1, at the first hospital, he received his first cycle of therapy, which included paclitaxel (500 mg), nedaplatin (198 mg), the anti–epidermal growth factor receptor (EGFR) monoclonal antibody nimotuzumab (200 mg), and sintilimab (200 mg).

On Day 2, he developed a scattered red rash distributed all over the body and abnormal liver function alanine aminotransferase (ALT) 329 U/L, aspartate aminotransferase (AST) 96 U/L, consistent with Grade 2 cutaneous and hepatic irAEs.

On Day 5, at our hospital, these symptoms resolved over the next several days following treatment with intravenous compound ammonium glycyrrhizinate and polyene phosphatidylcholine for hepatoprotection, along with oral antihistamines (cetirizine and fexofenadine) for the rash. On Day 11, based on the general understanding that chemotherapy can cause myelosuppression and lymphopenia, and with the intention of enhancing immune reconstitution, a single subcutaneous dose of Tα1 (1.6 mg) was administered. On Day 13 (within 48 h of Tα1 injection), the patient developed high‐grade fever (39°C–40.5°C) and chills. Empirical antibiotic therapy (cefuroxime, then levofloxacin) was ineffective. His condition deteriorated over the following days, with the emergence of facial rash and edema, chest tightness, and progressive hypoxemia.

**FIGURE 1:**
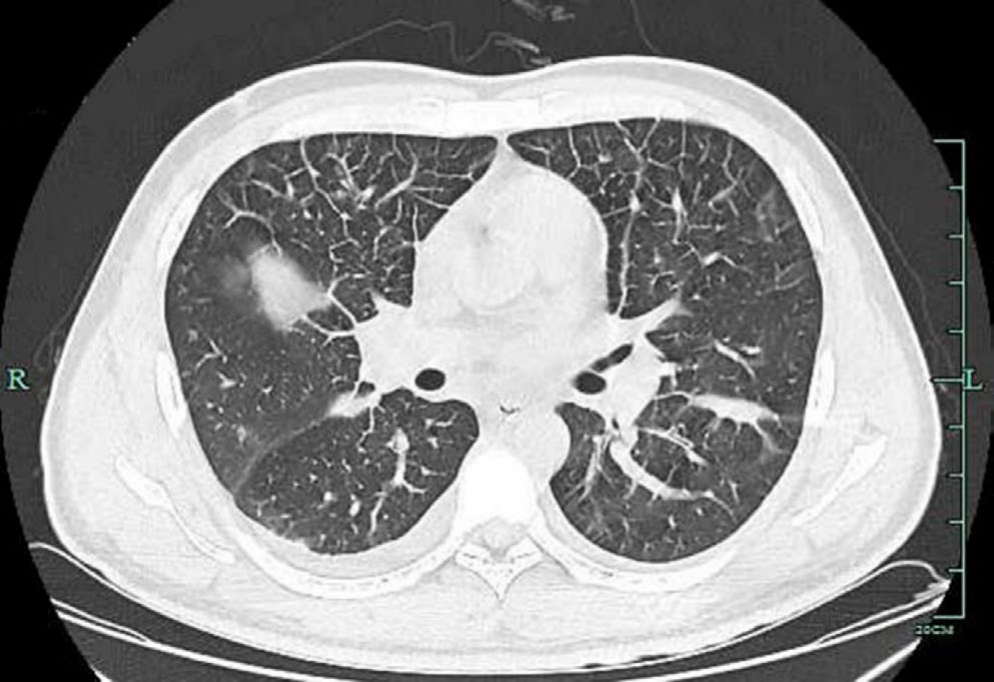
HRCT: Diffuse interstitial pulmonary edema and alveolar effusion in both lungs, with a small amount of fluid in the right pleural cavity.

On Day 17, a high‐resolution computed tomography (HRCT): Diffuse interstitial pulmonary edema and alveolar effusion in both lungs, with a small amount of fluid in the right pleural cavity (Figure 1). Laboratory tests: White blood cell (WBC) 8.46 × 10^9^/L, platelet count (PLT) 117 × 10^9^/L, C‐reactive protein (CRP) 70.48 mg/L, serum amyloid A (SAA) 323.34 mg/L, prothrombin time (PT) 23.2 s, PT activity 30.5%, procalcitonin (PCT) 11.430 ng/mL, partial pressure of oxygen in arterial blood (PaO₂) 72.8 mmHg, ALT 211 U/L, AST 742 U/L, gamma‐glutamyl transferase (GGT) 294 U/L. Given the lack of response to broad‐spectrum antibiotics (escalated to meropenem) and the explosive clinical course temporally linked to Tα1 administration, a diagnosis of severe, multisystem irAEs (including pneumonitis, hepatitis, dermatitis, and coagulopathy) triggered by the combination therapy was established.

On Day 21, the patient**'**s family requested transfer to Sir Run Run Shaw Hospital, affiliated with the Zhejiang University School of Medicine for further management.

## 3 Outcome and Follow‐Up

At the tertiary center, the primary diagnoses included multiorgan failure secondary to severe irAEs and possible sepsis. Laboratory tests: WBC 5.3 × 10^9^/L, PLT 67 × 10^9^/L, PT 17.4 s, PT activity 58%, PCT 11.07 ng/mL, PaO 51.6 mmHg, ALT 210 U/L, AST 956 U/L, and GGT 676 U/L. Management consisted of intravenous methylprednisolone at 160 mg daily (2 mg/kg) for immunosuppression, alongside continued broad‐spectrum antibiotics (meropenem and omadacycline) and comprehensive organ support. The patient responded rapidly to corticosteroids. The methylprednisolone dose was tapered according to the following schedule: 160 mg/day for 3 days, then 120 mg/day for 1 day, 80 mg/day for 2 days, 40 mg/day for 3 days, and 20 mg/day for 3 days. This constituted a total intravenous steroid course of 12 days.

On Day33, the patient was clinically stable and was discharged. This was followed by an oral methylprednisolone taper over an additional 2 weeks. No rebound of irAE symptoms occurred during the taper.

Adjusted strategy for subsequent antitumor therapy: based on the patient's poor tolerance of ICIs, the regimen was switched to chemotherapy (paclitaxel 420 mg plus nedaplatin 160 mg) combined with targeted therapy (nimotuzumab 200 mg) and definitive local radiotherapy to the nasopharyngeal and cervical regions (approximately 2–3 months after discharge). During radiotherapy, the patient received maintenance nimotuzumab (200 mg once weekly for six doses). Approximately 3–5 months after discharge, sintilimab was reintroduced at 200 mg every 3 weeks for six cycles; no further serious irAEs occurred, and the patient remained in stable disease.

## 4 Discussion

We describe a patient who developed marked systemic inflammation shortly after Tα1 administration in the setting of recent sintilimab exposure. The episode was characterized by high fever, diffuse rash/edema, hypoxemia with HRCT findings consistent with diffuse interstitial pulmonary edema/alveolar effusion, acute liver injury, and coagulation abnormalities. Infection and sepsis were considered and empiric broad‐spectrum antibiotics were started. However, clinical deterioration despite antimicrobial therapy and the prompt improvement after high‐dose methylprednisolone supported an immune‐mediated process compatible with severe irAEs [8].

ICIs may break peripheral tolerance and trigger inflammatory injury across multiple organs, presenting as irAEs [9]. Although multisystem irAEs are relatively uncommon, they have been reported and can be difficult to manage [10, 11] Tα1 is an immunomodulatory peptide that can influence innate and adaptive immune responses, including T‐cell/NK‐cell function and cytokine profiles [12, 13]. In oncology practice, Tα1 has been explored as an adjunct in ICI‐based regimens with the aim of modulating antitumor immunity [14, 15, 16, 17]. In this context, the close temporal association between Tα1 administration and symptom onset raises the possibility that additional immune stimulation may have contributed to excessive immune activation in a susceptible individual.

Available clinical reports combining Tα1 with PD‐1‐based therapy generally suggest acceptable tolerability without a clear overall increase in toxicity. For example, in unresectable hepatocellular carcinoma, adding Tα1 to lenvatinib plus sintilimab was reported not to significantly increase adverse events [18]. In a study including patients with advanced metastatic melanoma, the Tα1 combined with dacarbazine and interferon‐α treatment group had a longer overall survival than the control group [19]. A multicenter phase II study combining hypofractionated radiotherapy, a PD‐1 inhibitor, granulocyte‐macrophage colony‐stimulating factor, and Tα1 reported adverse events mainly including dermatologic reactions, thrombocytopenia, hypothyroidism, and fever [20]. Tα1 enhances the antitumor activity of CTLA‐4 and is also effective in preventing adverse immunotoxicity in the gut by promoting the indoleamine 2,3‐dioxygenase 1‐dependent tolerogenic immune pathway [21]. However, these data may not be designed to detect rare but severe events. Our case therefore adds clinically relevant caution that serious systemic inflammatory toxicity may still occur.

Therapeutically, corticosteroids have historically been effective in relieving irAEs, and their immunosuppressive effects directly counteract ICI‐induced widespread immune activation [22, 23]. In our patient, methylprednisolone led to rapid improvement, followed by tapering without rebound. The patient subsequently underwent sintilimab rechallenge without recurrence of severe toxicity. Although rechallenge can be feasible in selected patients after prior severe immune toxicity [24], it should be individualized and performed with close monitoring according to guideline recommendations.

Overall, this case highlights that, despite reassuring safety signals from published cohorts, severe systemic inflammatory toxicity may occur rarely when Tα1 is administered in proximity to PD‐1 blockade. Early recognition, exclusion of infection, and timely guideline‐based immunosuppression are essential to improve outcomes [23].

## 5 Conclusions

PD‐1 inhibitors combined with Tα1 may, in rare cases, be associated with severe multisystem irAEs, potentially through exaggerated immune activation. Clinicians should remain vigilant when considering this combination and carefully evaluate individual risk factors before use. For patients who develop severe irAEs, prompt interruption of ICIs and guideline‐based immunosuppressive management, with reassessment of the anticancer regimen, are essential. Further studies are warranted to define optimal patient selection, dosing, timing, and monitoring strategies to balance efficacy and safety when ICIs are combined with immunomodulators.

## Author Contributions

**Ting Li:** writing – review and editing. **Bao‐Liang Wu:** resources. **Cai‐Long Yu:** writing – review and editing. **Liang‐Yan Jin:** conceptualization, writing – review and editing.

## Funding

This research received no specific grant from any funding agency in the public, commercial, or not‐for‐profit sectors.

## Ethics Statement

This research has been approved by the Ethics Committee of Hangzhou Xixi Hospital, with the approval document number: 2025‐064.

## Consent

Written informed consent was obtained from the patient to publish this report in accordance with the journal's patient consent policy.

## Conflicts of Interest

The authors declare no conflicts of interest.

## Data Availability

Data sharing is not applicable to this article as no datasets were generated or analyzed during the current study.

## References

[1] Q. Liu , L. Li , W. Qin , et al., “Repurposing Drugs for Solid Tumor Treatment: Focus on Immune Checkpoint Inhibitors,” Cancer Biology & Medicine 20, no. 11 (2023): 856–868. 37929901 10.20892/j.issn.2095-3941.2023.0281 PMC10690875

[2] D. Y. Wang , J. E. Salem , J. V. Cohen , et al., “Fatal Toxic Effects Associated With Immune Checkpoint Inhibitors,” JAMA Oncology 4, no. 12 (2018): 1721–1728. 30242316 10.1001/jamaoncol.2018.3923 PMC6440712

[3] H. Yang , M. Sun , X. Zhou , et al., “Severe Immune‐Mediated Myocarditis Caused by Sintilimab Combined With Gemcitabine: A Case Report and Literature Review,” Frontiers in Cardiovascular Medicine 12 (2025): 1559173. 40271131 10.3389/fcvm.2025.1559173 PMC12014726

[4] L. Mao , “Thymosin Alpha 1 – Reimagine Its Broader Applications in the Immuno‐Oncology Era,” International Immunopharmacology 117 (2023): 109952. 36871535 10.1016/j.intimp.2023.109952

[5] E. Garaci , F. Pica , C. Matteucci et al., “ Historical Review on Thymosin α1 in Oncology: Preclinical and Clinical Experiences,” Expert Opinion on Biological Therapy 15, no. 1 (2015): 31–39. 10.1517/14712598.2015.1017466 26096345

[6] C. Costantini , M. M. Bellet , M. Pariano , et al., “A Reappraisal of Thymosin Alpha1 in Cancer Therapy,” Frontiers in Oncology 9 (2019): 873. 31555601 10.3389/fonc.2019.00873 PMC6742685

[7] R. S. Schulof , M. J. Lloyd , P. A. Cleary , et al., “A Randomized Trial to Evaluate the Immunorestorative Properties of Synthetic Thymosin‐Alpha 1 in Patients With Lung Cancer,” Journal of Biological Response Modifiers 4, no. 2 (1985): 147–158. 3998766

[8] J. Naidoo , C. Murphy , M. B. Atkins , et al., “Society for Immunotherapy of Cancer (SITC) Consensus Definitions for Immune Checkpoint Inhibitor‐Associated Immune‐Related Adverse Events (irAEs) Terminology,” Journal for Immunotherapy of Cancer 11, no. 3 (2023): e006398. 37001909 10.1136/jitc-2022-006398 PMC10069596

[9] J. A. Marin‐Acevedo , R. M. Chirila , and R. S. Dronca , “Immune Checkpoint Inhibitor Toxicities,” Mayo Clinic Proceedings 94, no. 7 (2019): 1321–1329. 31272574 10.1016/j.mayocp.2019.03.012

[10] G. Kichenadasse , J. O. Miners , A. A. Mangoni , A. Rowland , A. M. Hopkins , and M. J. Sorich , “Multiorgan Immune‐Related Adverse Events During Treatment With Atezolizumab,” Journal of the National Comprehensive Cancer Network 18, no. 9 (2020): 1191–1199. 32886899 10.6004/jnccn.2020.7567

[11] A. Laparra , M. Kfoury , S. Champiat , et al., “Multiple Immune‐Related Toxicities in Cancer Patients Treated With Anti‐Programmed Cell Death Protein 1 Immunotherapies: A New Surrogate Marker for Clinical Trials?,” Annals of Oncology 32, no. 7 (2021): 936–937. 33865965 10.1016/j.annonc.2021.04.006

[12] A. Dominari , D. H. Iii , K. Pandav , et al., “Thymosin Alpha 1: A Comprehensive Review of the Literature,” World Journal of Virology 9, no. 5 (2020): 67–78. 33362999 10.5501/wjv.v9.i5.67 PMC7747025

[13] Y. T. Wei , X. R. Wang , C. Yan , et al., “Thymosin α‐1 Reverses M2 Polarization of Tumor‐Associated Macrophages During Efferocytosis,” Cancer Research 82, no. 10 (2022): 1991–2002. 35364609 10.1158/0008-5472.CAN-21-4260

[14] K. Liu , L. Kong , H. Cui , et al., “Thymosin α1 Reverses Oncolytic Adenovirus‐Induced M2 Polarization of Macrophages to Improve Antitumor Immunity and Therapeutic Efficacy,” Cell Reports Medicine 5, no. 10 (2024): 101751. 39357524 10.1016/j.xcrm.2024.101751 PMC11513825

[15] R. Danielli , F. Cisternino , D. Giannarelli , et al., “Long‐Term Follow Up of Metastatic Melanoma Patients Treated With Thymosin Alpha‐1: Investigating Immune Checkpoints Synergy,” Expert Opinion on Biological Therapy 18, no. 1 (2018): 77–83. 10.1080/14712598.2018.1494717 30063847

[16] J. Galon and D. Bruni , “Approaches to Treat Immune Hot, Altered and Cold Tumours With Combination Immunotherapies,” Nature Reviews Drug Discovery 18, no. 3 (2019): 197–218. 30610226 10.1038/s41573-018-0007-y

[17] J. Nagasaki and Y. Togashi , “A Variety of ‘Exhausted’ T Cells in the Tumor Microenvironment,” International Immunology 34, no. 11 (2022): 563–570. 35460561 10.1093/intimm/dxac013

[18] S. Yao , Q. Huang , Y. Zou , et al., “The Efficacy and Safety of Thymosin Alpha‐1 Combined With Lenvatinib Plus Sintilimab in Unresectable Hepatocellular Carcinoma: A Retrospective Study,” Scientific Reports 15, no. 1 (2025): 13960. 40263352 10.1038/s41598-025-97160-7 PMC12015295

[19] M. Maio , A. Mackiewicz , A. Testori , et al., “Large Randomized Study of Thymosin α 1, Interferon Alfa, or Both in Combination With Dacarbazine in Patients With Metastatic Melanoma,” Journal of Clinical Oncology 28, no. 10 (2010): 1780–1787. 20194853 10.1200/JCO.2009.25.5208

[20] J. Yu , L. Yin , W. Guo , et al., “Hypofractionated Radiotherapy Combined With a PD‐1 Inhibitor, Granulocyte Macrophage‐Colony Stimulating Factor, and Thymosin‐α1 in Advanced Metastatic Solid Tumors: A Multicenter Phase II Clinical Trial,” Cancer Immunology, Immunotherapy 74, no. 3 (2025): 98. 39904914 10.1007/s00262-024-03934-9 PMC11794727

[21] G. Renga , M. M. Bellet , M. Pariano , et al., “Thymosin α1 Protects From CTLA‐4 Intestinal Immunopathology,” Life Science Alliance 3, no. 10 (2020): e202000662. 32817121 10.26508/lsa.202000662 PMC7441522

[22] N. B. Curkovic , R. Irlmeier , X. Bai , et al., “Impact of Steroid Dose and Timing on Efficacy of Combination PD‐1/CTLA‐4 Blockade,” Oncoimmunology 14, no. 1 (2025): 2494433. 40248956 10.1080/2162402X.2025.2494433 PMC12013437

[23] J. R. Brahmer , C. Lacchetti , B. J. Schneider , et al., “Management of Immune‐Related Adverse Events in Patients Treated With Immune Checkpoint Inhibitor Therapy: American Society of Clinical Oncology Clinical Practice Guideline,” Journal of Clinical Oncology 36, no. 17 (2018): 1714–1768. 29442540 10.1200/JCO.2017.77.6385 PMC6481621

[24] L. Ye , W. R. Yue , H. Shi , J. R. Li , and Y. Y. Qun , “Case Report: Successful Immune Checkpoint Inhibitor Rechallenge After Sintilimab‐Induced Guillain‐Barré Syndrome,” Frontiers in Immunology 16 (2025): 1546886. 40176803 10.3389/fimmu.2025.1546886 PMC11961408

